# Engaging Gypsy, Roma, and Traveller Communities in Research: Maximizing Opportunities and Overcoming Challenges

**DOI:** 10.1177/1049732318813558

**Published:** 2019-01-02

**Authors:** Louise Condon, Helen Bedford, Lana Ireland, Susan Kerr, Julie Mytton, Zoe Richardson, Cath Jackson

**Affiliations:** 1 Swansea University, Swansea, Wales, United Kingdom; 2 UCL Great Ormond Street Institute of Child Health, London, United Kingdom; 3 Glasgow Caledonian University, Glasgow, Scotland; 4 University of the West of England, Bristol, United Kingdom; 5 NHS Wakefield Clinical Commissioning Group, Wakefield, United Kingdom; 6 University of York, York, United Kingdom

**Keywords:** travellers, Gypsies, Roma, qualitative research, engagement, public involvement, vulnerable groups, research methods, qualitative interviews, United Kingdom

## Abstract

Gypsy, Roma, and Traveller people are marginalized worldwide and experience severe health inequalities, even in comparison to other ethnic minority groups. While diverse and hard to categorize, these communities are highly cohesive and members have a strong sense of identity as a group apart from the majority population. Researchers commonly experience challenges in accessing, recruiting, and retaining research participants from these communities, linked to their outsider status, insular nature, and history of discrimination. In this article, the challenges and the opportunities of engaging Gypsies, Roma, and Travellers in a multicenter qualitative research project are discussed. The management of public involvement and community engagement in this U.K.-based project provides insights into conducting research effectively with ethnically and linguistically diverse communities, often considered to be “hard to reach.”

## Introduction

Qualitative research necessitates the engagement of the community of study to attract sufficient participants who are willing to share their views freely with researchers. “Hard-to-reach” groups are defined as those who are socioeconomically disadvantaged and socially excluded and are least likely to be represented in research studies (Bonevski et al., 2014). Engaging people from Gypsy, Roma, and Traveller (Gypsy/Traveller) communities presents particular challenges in view of their long global history of genocide, banishment, discrimination, and rejection by mainstream society (Equality and Human Rights Commission [EHRC], 2016; Liégeois, 2007). These challenges are compounded by issues associated with language and literacy, travel and work commitments, and frequent mistrust of authorities (Kelleher, Whelan, Daly, & Fitzpatrick, 2011; Smith & Ruston, 2013). While often considered “hard to reach” by outsiders, Gypsy/Traveller communities are highly cohesive with a shared set of moral values and enduring family ties, and Liégeois (2007) suggests these strong internal social structures are the bedrock of their success in resisting eradication throughout history, and their survival as a distinct ethnicity.

The engagement of similarly socially excluded and stigmatized groups in research has previously been considered. Studies with relevance to Gypsy/Traveller research have focused on indigenous populations, people with mental health and substance use problems, disabilities, and low levels of literacy, and sex workers, who are also absent from many health records (Benoit, Jansson, Millar, & Phillips, 2005; Bonevski et al., 2014; Liljas et al., 2015). Review level evidence demonstrates that barriers to participation include difficulties in establishing a sampling frame and challenges in recruitment and data collection (Bonevski et al., 2014). Difficulties in establishing a sampling frame are often linked to researchers’ uncertainty about which people belong within a specified group (Benoit et al., 2005), as well as reluctance to “self-identify” with stigmatized others (Heckathorn, 1997). Barriers to recruitment arise from mistrust, fear of harm, and cultural beliefs (particularly in relation to sensitive health-related topics); additionally gatekeepers may make paternalistic judgments about the capacity of people from socially disadvantaged groups to participate in research (Bonevski et al., 2014). Problems associated with the process of data collection include language difficulties and low levels of literacy and education (Bonevski et al., 2014). To date, there has been little exploration of the opportunities, as well as the specific challenges, of engaging people from Gypsy, Roma, and Traveller communities in qualitative research.

Epidemiological studies of the health status of Gypsy/Travellers show poorer health and a higher risk of premature death than comparison groups matched by socioeconomic status (Parry et al., 2007; Peters et al., 2009; Van Cleemput & Parry, 2001). The reasons for these health inequalities are complex but include the stress associated with experiencing racism and discrimination (MacLachlan, 2006). Despite high health needs, uptake of health services, including preventive services, is poor (Cemlyn, Greenfields, Burnett, Matthews, & Whitwell, 2009; Peters et al., 2009). After the accession of Central and Eastern European countries to the European Union in 2004, the number of Roma people migrating to the United Kingdom increased. In common with U.K. Gypsy/Travellers, Central and Eastern European Roma experience discrimination, health inequalities, and barriers to uptake of health services, which exceed those of other minorities (Parekh & Rose, 2011; Zeman, Depken, & Senchina, 2003).

In 2012, the U.K. National Institute for Health Research (NIHR) called for qualitative research proposals to explore Travellers’ perspectives on immunization and to identify interventions to increase uptake. Immunization rates are low among Gypsies and Travellers (Dar, Gobin, Hogarth, Lane, & Ramsay, 2013; Feder, Vaclavik, & Streetly, 1993), with one study reporting the risk of measles as 100 times higher than in the general population (Maduma-Butshe & McCarthy, 2012). Factors affecting uptake of immunization services in the general population have been widely researched (Forster et al., 2016); however, much less is known about what influences uptake in Gypsy/Traveller communities, despite the higher risks of outbreaks of communicable disease (Dar et al., 2013; Newton & Smith, 2017).

In this article, we draw on our experiences of engaging and involving Gypsies, Roma, and Travellers in an NIHR funded study (the UNITING study). The study was conducted from 2013 to 2015 in four U.K. cities and included 174 interviews with community members (Jackson et al., 2017; Jackson et al., 2015; Jackson et al., 2016). Ethical approval was granted by the NRES Committee Yorkshire and the Humber-Leeds East (Ref. 13/YH/02) and consent was sought and received from all participants. This article explores the opportunities and challenges we encountered in relation to defining the study population, public involvement, gaining access, and recruiting participants.

## Defining the Study Population

### Categorization of Gypsies, Roma, and Travellers

Owing to the heterogeneity of Travelling people, a primary challenge is determining from which communities to recruit participants (Brown & Scullion, 2010). Among the range of people described as “Gypsy/Travellers” are English, Welsh and Scottish Gypsies, Irish Travellers, New Age Travellers, Boat People and Show People, as well as Roma from a variety of central and eastern European countries (Van Cleemput, 2010). Of these, only Gypsies and Irish Travellers were specifically included as an ethnic group in the 2011 U.K. Census, in which these two distinct groups were subsumed into one category, despite differences of language, history, and culture. In continental Europe, Roma people are no longer officially described as Gypsies as the word has become associated with racial abuse (Zeman et al., 2003). Many different groups are described as Roma (e.g., Manouches, Ashkali, Sinti, & Boyash people, European Commission, 2017), and a variety of dialects of the Romani language are spoken. Messing (2014) suggests that there are no objective criteria to determine Roma ethnicity, and as a whole, membership of the Gypsy/Traveller community is fluid rather than fixed, with some people “marrying into” the community and others ceasing to belong. Due to high levels of discrimination and prejudice, many members do not readily state their ethnicity to strangers or in bureaucratic situations (Equality and Human Rights Commission, 2016), which contributes to lack of knowledge about who self-identifies as a Gypsy, Roma, or Traveller.

In “outsider” research where the commissioners and lead researchers do not share the same ethnicity as participants, defining the study population is problematic (Brown & Scullion, 2010). Willems (1997) raises the question of the agent identifying the respondent and asks “Who defines who is a Gypsy?.” The UNITING study was designed to include multiple Gypsy/Traveller communities, including Occupational Travellers. Occupational Travellers who run fairs and circuses (known as Showpeople) are not part of the Census classification of Gypsies and Travellers; moreover, key informants suggested their attitudes and beliefs were likely to differ from other subgroups. In this way, “deviant cases” were purposively selected to extend the diversity of the sample (Ritchie, Lewis, & Elam, 2003). Pragmatic factors also played a part in defining the study population, such as which groups were well represented in the area, the existence of organizations with whom researchers could liaise, and researchers’ prior research links with local communities.

### Modification of the study population

High levels of flexibility are required of researchers working with socially and ethnically complex, and disadvantaged populations. In the course of this project, a number of changes were made to the ethnic, national, and age composition of proposed sample in response to local and national factors. The aim was to include Roma from continental Europe because of the heightened disadvantage experienced by these recent migrants to the United Kingdom (Brown & Scullion, 2013; Burchardt, Obolenskaya, Vizard, & Battaglini, 2018). The Roma component of the sample increased when numbers of Romanian Roma moving to the United Kingdom multiplied in 2014, due to changes in EU right to work legislation. Consequently, the final sample was extended to include Romanian Roma in two study sites as well as Slovakian Roma participants.

Local factors also resulted in variation in the planned and actual study population, demonstrating the potency of outside influences on sample selection. Where English Gypsies were the planned sample, one Gypsy gatekeeper requested that Irish Travellers who lived as neighbors on the same local authority sites were offered the same opportunity to participate. This gatekeeper considered that as a worker employed to represent the whole community she must be seen to be impartial, and to favor one group above another would jeopardize her position, even her future advocacy role. Here distinction could not be made between subgroups without causing offense to the community and risking the engagement of an influential gatekeeper.

Liamputtong (2010) suggests that a core challenge of cross-cultural research is “placing” potential participants within an ethnic or cultural group to decide their eligibility to be included. “Placing” is an uncomfortable activity as it requires researchers to consider a potential participant from outside and make judgments about their identity and group membership. In a development of this concept, it was apparent from this study that gatekeepers who themselves belong to the community of study, experience challenges in “placing” participants. Anomalies of “placing,” seemingly linked to fear of discriminating unfairly between individuals, also led to gatekeepers recruiting a small number of people who did not consider themselves ethnic Gypsy/Travellers, but who had “married into” the community and were therefore considered as members. Thus, sensitivity to the opinions and allegiances of gatekeepers, responsivity to political and social factors, and flexibility within the inclusion criteria specified had a tangible effect upon the defined population of study, rendering it more heterogeneous and fluidly defined than initially planned. Table 1 shows the range of communities which were sought and which were ultimately achieved.

**Table 1.** Planned and Actual Sample by Ethnicity and Nationality in Each Study Site.

	Site 1	Site 2	Site 3	Site 4
Planned	1. Romanian Roma 2. English Gypsy	English Roma	1. Slovakian Roma 2. Scottish Showpeople	Irish Traveller
Actual	1. Romanian Roma 2. English Gypsy 3. Irish Traveller	English Roma (but included within the English Gypsy categorization as they agreed they belong to the same ethnic group)	1. Slovakian Roma 2. Scottish Showpeople 3. Romanian Roma	Irish Traveller

## Public Involvement

Comprehensive and well-conducted public involvement is vital to ensure the relevance and acceptability of research and most important in communities with a heightened distrust of outsiders. Public involvement has been defined as research being carried out “with” or “by” members of the public rather than “to,” “about,” or “for” them (INVOLVE, 2016). Immunization among Traveling communities was identified by the U.K. NIHR as a public health priority, rather than arising from an explicit need expressed by the people themselves—thus the approach was initially “top down.” However, this did not prove a barrier to community members joining the research team or taking part in consultation events with representatives from the wider Gypsy/Traveller community.

In the UNITING study, public participation did not extend to identification of the initial research priorities or selection the topic of study; however, two Gypsy/Traveller research team members contributed to research design and study delivery, with one also conducting some interviews (Jackson et al., 2016). This high level of engagement is likely to be linked to the acceptability of the topic, a view supported by study findings which showed no generalized cultural antipathy to vaccination but instead revealed barriers to accessing services (Jackson et al., 2017). Additional factors which contributed to successful public involvement were widespread recognition of health inequalities and existing relationships with researchers and/or health professionals at a local level. Interest in the topic of study and good researcher–community relationships are recognized as facilitators to successful engagement with marginalized groups (Liamputtong, 2007).

### Community Partnership

Gypsy/Traveller research team members had experience of working in advocacy posts (local government and third sector) and contributed their inside knowledge to the otherwise non-Gypsy (“gadje”) research team. While the research team members were themselves atypical of the community as a whole (e.g., in being literate and accustomed to working with the “gadje”), they contributed to facilitating wider public involvement in the form of local Community Partnership groups, which drew from the wider community. As de Freitas and Martin (2015) suggest, marginalized groups need to build confidence, capacity, and a sense of entitlement to practise their citizenship and exploit opportunities for participation, and local groups served to extend the diversity of public involvement. Community Partnership groups met 4 to 5 times in each study center during the course of the 2-year project, providing research guidance at a local level.

Barnes, Newman, Knops, and Sullivan (2003) remind us that the “public” are not a single group who share a collective identity, a highly salient point in relation to GRT communities. In two of the four study centers, the planned target sample included Roma and non-Roma communities, which increased the complexity of public involvement due to cultural and linguistic differences. It is recognized that there is no requirement for Community Partners to be representative of the whole community (Staniszewska, Haywood, Brett, & Tutton, 2012), but to maximize involvement representation was sought from both Roma and non-Roma. When joint Community Partnership meetings were attempted, Roma people were less likely to attend and contribute, even if they spoke good English. When a conscious effort was made to bring together English Gypsies and Roma people in equal numbers, with a trusted interpreter who was experienced in concurrent translation and bilingual group facilitation, enhanced engagement of the Roma community was achieved. This study was unusual in bringing together Community Partners who belonged to the same broad ethnic group but did not share a common language.

### The Role and Influence of Community Partners

The views of Community Partners were sought on such issues as the local terminology for vaccinations (variously “jabs,” ‘jags’ and “needles”), participant information materials, recruitment, data collection, and the dissemination of study findings. Facilitating discussion on the research process with people unused to abstract discussion presented a challenge, and highlighted difficulties in finding a common language between researchers and the public (Staniszewska et al., 2012). Where the purpose of a Community Partnership meeting was more abstruse, such as suggesting how the findings from interviews with Travellers could be used to stimulate discussion in interviews with service providers, the tendency was to revert to talk about personal experience of immunization. Meetings with Community Partners worked best when there was a clear agenda and pragmatic tasks to be accomplished. In view of low literacy and educational levels among Gypsy/Travellers (Liégeois, 2007; Van Cleemput, 2010), meetings were carefully planned to focus on oral rather than written group exercises, to avoid the potential embarrassment of exposing illiteracy. Using this approach, local information was obtained about the most suitable pictures and wording for posters and information sheets, and appropriate venues for data collection (Jackson et al., 2016).

In addition to these expected and traditional aspects of public involvement, Community Partners influenced the study in ways not anticipated by researchers. Taboos about premarital sexual activity led to one partnership group advising that HPV vaccination (which is given in the early teenage years to reduce the risk of cervical cancer when a woman becomes sexually active) should not be discussed with teenagers under 18 years of age. Allowing women younger than 18 years to participate in the study was stated to be unacceptable to men within the community who would interpret this as a slur upon their daughters’ purity. As no men were present this view could not be checked. However, as a consequence in this site only women aged 18 years or older took part, in contrast to other study sites where young women aged 16 years and older were free to participate—and hence had the opportunity to put forward their view on HPV vaccination, an issue of high relevance to the health of young people. This incident highlights an ethical dilemma of participation, by which “voice” is given to those whose views which do not necessarily concur with those of the research team, or even all sectors of the community, and which potentially denies “voice” to the those who have less power. Rowa-Dewar et al. (2008) note that health promotion is not within the remit of the researcher, but that attitudes to “correct” health-related beliefs are a potential source of tension between researchers and participants.

## Gaining Access to Participants

### The Enhanced Role of Gatekeepers

Social isolation and traditionally low levels of trust in outsiders result in Gypsy/Traveller communities being viewed as “highly closed” (Liamputtong, 2007). It is well recognized that only gatekeepers who are widely trusted and committed to a project can successfully facilitate community participation and negotiate access for researchers (Wanat, 2008); this is of paramount importance when the community to which access is being sought is marginalized (Smith, 2008). In the UNITING study, a range of gatekeepers were successfully identified, including members of the community, health professionals with specialist Traveller roles, and interpreters. Gatekeepers informed potential participants about the project, distributed written information and, in some communities, arranged times for a researcher to visit the selected venue and conduct interviews. Read and Maslin-Prothero (2011) highlight the importance of mutual respect and partnership working in research with vulnerable groups; where preexisting collaborative relationships between researchers and gatekeepers existed, this contributed to trust, facilitating access to potential participants. Only one gatekeeper was seemingly “protective” of participants in accompanying researchers to all interviews, whether in the community center or in participants’ homes.

Most gatekeepers found it easy to inform potential participants about the proposed research as part of their usual interaction with the community and to form a conduit between researchers and potential participants. This ease of access challenges the common stereotype of Gypsy/Travellers as a “hard to reach” group, which as Brown and Scullion (2010) make clear can be an overly convenient label used by researchers who lack experience and knowledge. Paradoxically it may be that in a group set apart from the majority population (geographically, ethnically, and socially) trusted gatekeepers can hold almost disproportionate power and influence, which facilitates involvement in research once links have been made between researchers and local gatekeepers.

In written information and in verbal introduction to the project by gatekeepers emphasis was put on “telling your story,” an approach which has been successful in previous projects (Condon & Salmon, 2015). This approach seeks to be nonthreatening and to reassure participants that their experience is of value and will be respected. Brown and Scullion (2010) have highlighted the potency of “being heard” for marginalized and socially excluded Gypsy/Travellers. High levels of sociability, and social cohesion within this community, meant that news of the project spread fast by word of mouth, often leading to enthusiasm to participate. All gatekeepers were part of the day-to-day social world of participants and could facilitate this process. Qualitative research may be popular among this highly oral culture, as people are used to engaging in lively conversation on a daily basis due to close family networks and prioritization of social interaction (Kiddle, 1999).

### Political Influences on Access

Changes to local or national policy and to legislation which arise during the lifetime of a study can have a powerful impact upon its conduct. Such changes may influence the engagement of members of the research team and their ability to access, recruit, and retain study participants. During our study, the 2012 Health and Social Care Act devolved decision making for health and social care to General Practitioners in England, and subsequently local authorities experienced funding cuts from national government. This meant that local authority funded posts were lost, including those which facilitated engagement, such as a Gypsy and Traveller Team, and an Equality and Diversity team. Brown and Scullion (2013) comment that reduced capacity, at a time of rising demand, results in loss of institutional memory about effective engagement—this has an impact upon research as well as practice. In this project, the challenge was partially addressed by former local authority employees continuing to be members of the study team in a freelance capacity, thus ensuring continuity of the trusted gatekeeper role, but overall there was a reduction in the availability of gatekeepers throughout the study. A second contributing factor to gatekeeper impermanence is that highly qualified Eastern Europe migrants may initially use their language skills in working with Roma people, but access to more privileged social networks (Ryan, 2011) can soon lead to more secure and prestigious work or study. This also had an impact on gatekeeper retention in this project.

## Recruitment

Recruitment strategies were derived from advice from Community Members, the prior experience of researchers, and evidence from academic literature (see Bonevski, 2014). These strategies were also discussed with a study advisory group made up of researchers with experience in conducting research with socially excluded groups. Of primary interest in qualitative research is whose voices are heard and whose are silenced (Kristensen & Ravn, 2015). When recruiting from “hard to reach” communities, it is almost inevitable that those who make most use of advocacy, health, and welfare services are most likely to be recruited, especially as gatekeepers are often drawn from these sectors.

### Including the Most Marginalized

The extent to which gatekeepers influenced the decision to participate is unknown, most notably in the case of Roma communities where few participants spoke English. Interpreters acting as gatekeepers had experience of interpreting for Roma people and existing good relationships with the community; however, none were themselves from a Roma Gypsy background due to the lack of Romani-speaking interpreters. Thus, interviews were conducted in participants’ second language in place of their Romani mother tongue. The part played by interpreters in qualitative research is now being acknowledged (Liamputtong, 2010; Temple & Edwards, 2002), but little critical focus has been directed toward the use of interpreters to enable community engagement in research. Those who work with Roma communities are in a unique situation due to the level of isolation and discrimination experienced by Roma people in their countries of origin (European Commission, 2017; European Union Agency for Fundamental Rights, 2014), and it is probable that the power differential between non-Roma interpreters and Roma participants influences participation.

Conducting interviews in participant’s homes or at community venues inevitably led to the inclusion of more settled members of the community, and no roadside Gypsy/Travellers or people living on unauthorized sites were represented in the final sample. While these groups were not consciously excluded, it is apparent that more specific measures would have been needed to attract the most marginalized. This could involve targeting transit sites which offer temporary accommodation for Gypsy/Travellers but transit sites are not consistently occupied and local gatekeepers are less likely to have established relationships with those who use them. This therefore presents additional challenges to recruitment and potentially adds to time pressures and project costs, substantiating the view that securing the voice of the most geographically and socially isolated demands high costs in time and effort (Thompson & Phillips, 2007).

### Rewarding Participation

Using incentives to foster research participation is a highly debated issue. From an ethical perspective, some suggest that offering any form of recompense influences an individual’s choice to participate by providing an additional inducement (Grady, 2005). Others maintain that offering a financial “thank you” partially redresses the inequality in power between paid researchers and volunteers (Hammett & Sporton, 2012). It is undeniable that in poorer communities recompense may act as a more powerful incentive than elsewhere, and for this reason, Grant and Sugarman (2004) have suggested that it is hence ethically unwise to offer financial recompense. In this study, a supermarket voucher was offered as a “thank you” for participants’ time, which was mentioned by those recruiting to the study and included in the information leaflet. Gatekeepers, collaborators, and Community Partners identified the provision of vouchers as being an important factor in attracting participants, although not equally successful in all subgroups. Despite fewer barriers to research participation in terms of literacy and language, it was not possible to recruit to the target sample of Showpeople. Some Showpeople expressed reservations about participating in a study which viewed their needs in relation to immunization as separate from the majority population (Jackson et al., 2016).

Successful recruitment by gatekeepers in combination with the provision of a “thank you” voucher led to greater than anticipated numbers of potential participants presenting for interview at “drop in” interview sessions in community venues. This resulted in some disruption of activities which were taking place concurrently, such as welfare advice sessions; for this reason specific appointments for targeted individuals were introduced. When interviewing in the home, pressure to interview all family members had to be balanced against recruiting to the predetermined criteria. On a small number of occasions, researchers felt that they had no choice but to include a participant who was not required for the sample; examples were a teenage boy who had been told by his mother to take time out of school to attend, and a young adult with disabilities and little speech who was present while a parent was being interviewed. The sample size was increased (see Table 2) to ensure that the proportion of certain groups (such as grandparents) was aligned to the planned sampling framework. This approach contributed to our achievement of exceeding our minimum recruited sample from all but one community.

**Table 2.** Planned and Actual Sample by Sex and Family Role Within Each Gypsy, Roma, and Traveller Community

	Fathers	Grandfathers	Adolescents/Young women	Mothers	Grandmothers	Male no children
Planned (range)	2–4	2–4	6–8	6–8	6–8	0
Actual (range)	0–6	0–2	2–11	9–19	3–8	0–4

## Discussion

The opportunities inherent in engaging people from “hard to reach” groups in research are rarely described. It is by building on these opportunities that challenges to engagement, access, public involvement and recruitment can be overcome. Partly as a result of the isolation of many Gypsy/Traveller communities, gatekeepers exist who have privileged access to the community and can assist researchers with access, whether community members or associated workers (Brown & Scullion, 2010). In this study, gatekeepers could relatively easily be found who were accustomed to forging links between communities and professionals. As a result of previous research and health improvement initiatives, some researchers, gatekeepers, and interpreters were known to each other in advance of the project which facilitated good working relationships. Flory and Ezekiel (2004) emphasize the importance of extended discussion and one-to-explanation to engage “hidden populations,” and gatekeepers to marginalized communities are well placed to perform this role. In a community with a strong oral tradition and love of lively debate and conversation (Kiddle, 1999), participants were ready to “tell their stories” about their personal experiences of immunizations and to speak from the perspective of their acknowledged and distinctive ethnic perspective and identity.

Although this public health topic had not been identified as a priority by Gypsy/Travellers themselves, widespread community-wide recognition of the injustice they experience meant that participants were keen to engage in this project, a point noted in relation to other vulnerable populations (Liamputtong, 2007). Once engaged in the research project, individuals were ready to suggest others who might wish to be involved to make a difference to health and health care, addressing persistent health disadvantage. “Thank you” vouchers act as a powerful incentive to participate in communities experiencing disadvantage and poverty, and therefore careful consideration must be given to how, when, and where vouchers are administered. Bancroft (1997) suggests that those who are “double stigmatized" (he gives the example of men who are substance misusers and have sex with men) are less likely to participate in research. In the study, Roma people, who are both Gypsies and recent migrants, could be considered as “double stigmatized,” but the measures described above were sufficient to ensure the target numbers of Roma participants were reached.

Challenges to engagement of “hard to reach” groups in research predominantly resemble the challenges to service use, and are low literacy, lack of formal education, transient and precarious lives, anticipation of discrimination, and rejection (Bonevski et al., 2014). Often there are no gatekeepers for the most marginalized, such as roadside Travellers or those living on unauthorized sites, leading to inability to access these minorities (Brown & Scullion, 2010). New challenges identified in this study center on the gatekeeper role and its inherent complexities. These complexities vary according to whether gatekeepers are members of the community of study or “gadje.” When non-Roma interpreters are used as gatekeepers to the Roma community, barriers to open communication may exist, which are not easily apparent to researchers. These include lack of a shared mother tongue and differences of status, which potentially influence engagement in unforeseen ways. The health needs of migrant people are profoundly affected by such factors as nationality, socioeconomic circumstances, religion, and length of residence (Gazard, Frissa, Nellums, Hotopf, & Hatch, 2015; Jayaweera & Quigley, 2010), and it is apparent that similar factors influence the engagement of Roma people in research.

For gatekeepers of Gypsy/Traveller ethnicity, there are pressures associated with keeping faith with the community, for instance by not favoring one ethnic community above another in recruitment, while complying with researchers in meeting the exigencies of a tightly defined and predetermined research protocol (Aldridge, 2014; Bonevski et al., 2014). Most centrally, the diversity and fluidity of the Gypsy/Traveller identity can lead to difficulty in “placing” individuals as community members, even when gatekeepers are themselves members of the community. Not all individuals will necessarily accept classifications imposed from outside, as exemplified in this study when Showpeople resented being identified with other subgroups (such as Roma people), seeing themselves as closely aligned to the general population in their practices and beliefs about immunization. When presenting study findings, care is needed in not assuming and presenting an overly homogeneous “voice of the community.” Wiebel (1990) uses the term “hidden population” of those who have poorly defined membership of a somewhat amorphous group, and this study has demonstrated that while Gypsy/Traveller ethnicity is strongly evident both within and outside the community, there is a level of heterogeneity which is rarely explored.

This study has demonstrated that challenges and opportunities do not remain static throughout a long-term project as they are subject to national and local policy changes. During the course of this study, readjustment of the sample was made when new Roma groups migrated to the United Kingdom. Reductions in local authority budgets led to a decrease in the availability of established gatekeepers in some study sites. Relationships of trust with gatekeepers and researchers are a prerequisite of engaging marginalized communities in research and where cuts are made to targeted services the ability to involve Gypsy/Traveller people in research is reduced.

## Conclusion

This study adds to knowledge of engaging culturally and linguistically diverse ethnic minority groups in research, an area which is currently under researched. It has raised new issues such as how voice can be denied or granted within Gypsy/Traveller communities and the power differentials between Roma participants and interpreters who share the same nationality but not ethnicity. It has highlighted the tightrope walked by gatekeepers who must balance “keeping faith” with their own ethnic group (and other subgroups), with facilitating access for researchers. It has confirmed that strategies such as adopting a nonthreatening and respectful approach, ensuring that recruitment materials are accessible to participants, conducting interviews in familiar places and offering a “thank you” voucher contribute to effective engagement. When engaging with vulnerable and disadvantaged populations, high levels of resilience and flexibility are required of researchers, and there is a high likelihood of strategic readjustment being needed during the course of the project. Such issues need to be taken into account when planning a study with “hard to reach” groups, and it is essential that the additional time and costs associated with engagement of the most marginalized are included in the budget and justified to commissioners of research.
